# ELDER CARE COMPLEXITIES AND OUTCOMES: A MANDATE FOR INTERDISCIPLINARY GERIATRIC CLINICAL ASSESSMENT

**DOI:** 10.1093/geroni/igz038.1857

**Published:** 2019-11-08

**Authors:** Peter S Reed, Zebbedia Gibb

**Affiliations:** Sanford Center for Aging, University of Nevada Reno School of Medicine, Reno, Nevada, United States

## Abstract

Interdisciplinary geriatric assessment has long been considered best practice to identify the full range of elder care needs. In 2015, the Sanford Center for Aging launched an interdisciplinary comprehensive geriatric assessment clinic. This team-based assessment includes a geriatrician, social worker and pharmacist meeting together with each client to review all aspects of their health and well-being, resulting in a comprehensive care plan to coordinate care with the client’s primary care provider. To assess this approach, a survey was conducted with 415 randomly-selected clients prior to the clinical visit, with a 6-month follow-up survey completed for 170 clients (41%), gathering data on hospitalizations, long-term care utilization and quality of life. Combining these data with clinical assessment data provides a picture of clinical complexities of elder clients and offers a mandate for comprehensive interdisciplinary care. Baseline data showed 44% hospitalized in the prior year; an average of 4 chronic conditions and 10 medications; 44% with dementia or MCI and 29% with frailty. At 6-months post-assessment, 29% reported being hospitalized, 3.5% reported moving into long-term care, and there was a slight, non-significant decrease in quality of life. These data demonstrate the profound complexities that can be identified and addressed through comprehensive assessment and care, as well as the potential to reduce hospitalizations, enable people to age-in-place and maintain quality of life. Despite the well-documented value of these approaches, Medicare and other payers have not fully embraced the opportunity to achieve these positive outcomes and remain hesitant to adequately fund comprehensive approaches to care.

